# Evaluation of sleep characteristics of children and adolescents with type 1 diabetes mellitus

**DOI:** 10.1590/1984-0462/2022/40/2020407

**Published:** 2021-10-04

**Authors:** Renata Aparecida e Silva, Aline De Piano Ganen, Vânia de Fátima Tonetto Fernandes, Nara Michelle de Araújo Evangelista, Carolina Costa Figueiredo, Luciana de Aguiar Pacheco, Guido de Paula Colares

**Affiliations:** a Centro Universitário São Camilo, São Paulo, SP, Brazil.; b Hospital Infantil Darcy Vargas, São Paulo, SP, Brazil.

**Keywords:** Diabetes mellitus, type 1, Glycated hemoglobin, Sleep, Children, Adolescents, Diabetes mellitus tipo 1, Hemoglobina glicada, Sono, Crianças, Adolescentes

## Abstract

**Objective:**

To evaluate sleep characteristics of children and adolescents with type 1 diabetes mellitus (T1DM) and their relationship with glycemic control.

**Methods:**

A cross-sectional study was conducted at a public hospital in São Paulo, Brazil. It included 86 patients with T1DM, aged between 10 and 18 years old, who were on insulin therapy, had performed at least three measurements of capillary blood glucose throughout the day, and had normal thyroid function. The clinical, anthropometric, and laboratory data of each patient were evaluated. The Pediatric Daytime Sleepiness Scale (PDSS) and the Munich Chronotype Questionnaire (MCTQ) were used to assess the sleep characteristics.

**Results:**

The mean level of glycated hemoglobin (HbA1c) was 9.2±2.1%, and it was higher in adolescents than in children. The mean score of PDSS was 13.9±4.7. Patients with HbA1c<7.5% had lower PDSS scores and longer sleep duration on weekdays than patients with HbA1c≥7.5%. HbA1c levels were negatively correlated with chronotype values and sleep duration on weekdays and positively correlated with social jet lag. Patients who had had T1DM for less than three years had a higher prevalence of daytime sleepiness. The regression analysis showed that higher HbA1c (≥7.5%) and shorter time since the diagnosis of T1DM increased the chance of daytime sleepiness, regardless of age and sex.

**Conclusions:**

Patients with higher HbA1c had more daytime sleepiness, a morning chronotype, shorter sleep duration on weekdays and a more significant social jet lag. The shorter diagnosis time for T1DM and greater levels of HbA1c increased the chance of daytime sleepiness.

## RESUMO

**Objetivo:**

Avaliar as características do sono em crianças e adolescentes portadores de diabetes melito tipo 1 (DM1) e sua relação com o controle glicêmico.

**Métodos:**

Estudo transversal realizado em um hospital público de São Paulo. A amostra foi composta de 86 portadores de DM1 entre 10 e 18 anos, aderentes à insulinoterapia, com monitoração mínima de três glicemias capilares ao dia e função tireoidiana normal. Foram avaliados os dados clínicos, antropométricos e laboratoriais de cada paciente. Utilizaram-se a Escala de Sonolência Diurna Pediátrica (ESDP) e o Questionário de Cronotipo de Munique (QCTM).

**Resultados:**

A média de hemoglobina glicada (HbA1c) foi 9,2±2,1%, sendo maior em adolescentes. A média do escore da ESDP foi 13,9±4,7. Pacientes com HbA1c<7,5% tiveram menor escore na ESDP e maior duração do sono em dias de semana do que pacientes com HbA1c≥7,5%. Verificaram-se correlações negativas da HbA1c com valores do cronotipo e com duração do sono em dias de semana e correlação positiva da HbA1c com *jet lag* social. Pacientes com tempo de DM1 menor que três anos tiveram maior prevalência de sonolência diurna. A análise de regressão apontou que, quanto maior a HbA1c e menor o tempo de diagnóstico de DM1, maior a chance de sonolência diurna, independentemente de idade e sexo.

**Conclusões:**

Pacientes com HbA1c mais elevada apresentaram mais sonolência diurna, cronotipo matutino, menor duração do sono em dias de semana e maior *jet lag* social. O menor tempo de diagnóstico de DM1 e HbA1c≥7,5% aumentaram a chance de maior sonolência diurna.

## INTRODUCTION

Type 1 diabetes mellitus (T1DM) is a chronic disease characterized by deficiency in insulin production due to the loss of pancreatic β cells, with consequent hyperglycemia. The prevalence is higher in the pediatric age group, and its annual incidence in Brazil among children aged 0 to 14 years is 7.3 cases/1,000.^1^ ^,^ ^2^

In the pediatric age group, patients with chronic diseases, including T1DM, are at greater risk of presenting sleep-related problems—such as daytime sleepiness, insufficient sleep and social jet lag—than healthy children. Children with T1DM are susceptible to sleep disorders because of the effect of glucose and insulin processing on the central nervous system and increased response to bodily stress.^3^ ^,^ ^4^

The interactions between sleep and T1DM are complex and bidirectional; impaired sleep can affect glycemic control, and uncontrolled blood glucose can interfere with sleep.^5^ In addition, nocturnal hypoglycemia and the need for immediate care can affect time spent in bed or sleep duration.^5^ ^,^ ^6^

Due to this possible interrelation between glycemic control in patients with T1DM and sleep quality, the aim of this study was to evaluate the association between glycemic control and sleep characteristics in children and adolescents with this condition.

## METHOD

The research was submitted to the Research Ethics Committee of Centro Universitário São Camilo and approved under the Certificate of Presentation of Ethical Appreciation (CAAE) 17967219.2.0000.0062 and opinion number 3.592.076.

Among the 249 patients with T1DM treated at the pediatric endocrinology outpatient clinic at Hospital Infantil Darcy Vargas in São Paulo, SP, 86 children (10 to 12 years old) and adolescents (13 to 18 years old) were selected and divided by age group according to the Statute of Children and Adolescents. The inclusion criteria were:

- having T1DM with adherence to insulin therapy;
- performing capillary blood glucose monitoring at least three times a day;
- having normal thyroid function.

The following exclusion criteria were used:

1. having associated autoimmune diseases, such as decompensated Hashimoto's thyroiditis and celiac disease;
2. having neurological disorders with the use of anticonvulsants;
3. being under treatment for sleep disorders.

Data collection was carried out from September 2019 to June 2020. The research and the free and informed consent were presented to children's/adolescents’ guardians, and the informed assent form to children and adolescents. After agreeing in participate, data were collected and the study questionnaires were applied.

The variables searched for in medical records were:

- chronological age;
- sex;
- pubertal staging, according to Marshall's and Tanner's criteria;^7^
- time of diagnosis of T1DM;
- insulin treatment (multiple doses of insulin [MDI] or continuous subcutaneous insulin infusion [CSII];
- glycemic control (number of capillary blood glucose measurements during the day and night).

For anthropometry, the most recent measurements of weight, in kilograms, height, in centimeters, and body mass index (BMI) were collected. Classifications of the Z score for height for age and BMI for age were performed based on reference data from the World Health Organization (WHO).^8^

Information about glycated hemoglobin (HbA1c) for the three months prior to the study was also collected.

Two questionnaires were used to assess sleep characteristics in all participating children and adolescents: the Pediatric Daytime Sleepiness Scale (PDSS), and the Munich Chronotype Questionnaire (MCTQ).

The PDSS is an eight-item questionnaire used to measure excessive sleepiness in school-age children and adolescents, validated and translated into Portuguese.^9^ ^,^ ^10^ Patients with an PDSS score greater than or equal to 15 are considered to have excessive daytime sleepiness, while a score below 15 indicates no excessive daytime sleepiness.^11^

The MCTQ was used to assess sleep patterns, habits and hours. It has also been validated and translated into Portuguese.^12^ ^,^ ^13^ It consists of questions related to the chronotype, the latency of night sleep (estimated time to fall asleep), the means used to wake up (spontaneously, alarm clock or someone calling), the total time in bed and sleep efficiency (total sleep time over total bed time), for both school days and weekend days. The chronotype is obtained by the midpoint of sleep on weekend days (MSF). MSF is then corrected for sleep debt on school days, giving rise to MSFsc. The classification of the chronotype is based on the MSFsc, according to the classification in the population of interest. Smaller values characterize the morning pattern and larger values of the sample, the afternoon pattern.

Social jet lag is the result of the difference between the average sleep period of weekend days and weekdays. It is indicative of the pressure on sleep phase to which the individual is exposed when this period is different from his endogenous sleep schedule.^12^

For the descriptive analysis of quantitative variables, central tendency measures were used, such as mean and minimum and maximum values; standard deviation was used as dispersion. With regard to categorical variables, frequency measures (absolute and relative) were calculated.

To check the magnitude of association between categorical study variables, the χ2 test was used, considering p≤0.05 significant. Then, variables with p<0.20 were taken into account in the univariate analysis, which, in ascending order of entry, made up the final model of multiple logistic regression. In this model, variables with p<0.05 remained, or those that changed the Odds Ratio (OR) of the variable of interest by at least 10%. It is noteworthy that sex and age were maintained to adjust the model, regardless of the p-value. The normality distribution of the data was verified using the Kolmogorov-Smirnov test. To compare means, the Student's t test for independent samples and the Mann-Whitney and Kruskal-Wallis tests were used when data were nonparametric. Correlations between the variables were made using the Spearman's and Pearson's tests, according to the normal distribution. Statistical data were performed using the software Stata version 13.0 for Windows.

## RESULTS

Among the 86 participants, 51.2% were females. Most were adolescents, 88.4% being in the pubertal phase. The mean age was 13.2±2.2 years; the mean time of T1DM was 5.4±3.7 years; and the mean HbA1c was 9.2±2.1%. Most of them (84.9%) were undergoing treatment with multiple insulin doses, and the mean daily capillary blood glucose was 5.5±1.7 (data not reported in the table).

All patients were classified with a Z score of height appropriate for their age. Regarding the BMI Z score, 63.9% were eutrophic and 34.9% were overweight or obese.

The mean values for the classification of sleep characteristics are described in Table 1.

**Table 1:** Mean values of variables related to sleep characteristics in children and adolescents. São Paulo, SP, Brazil, 2020.

		Mean±SD
Pediatric Daytime Sleepiness Scale Score (n=86)		13.9±4.7
	<15 (n=49)	10.9±3.4
	≥15 (n=37)	17.9±3.1
Munich chronotype (n=86)		4.1±0.8
	Tertile 1 (n=29)	3.8±0.5
	Tertile 2 (n=29)	4.2±0.2
	Tertile 3 (n=28)	4.9±0.5
Social jet lag (h) (n=86)		0.7±0.9
Sleep duration on weekend days (h) (n=86)		9.2±1.5
Sleep duration on weekdays (h) (n=86)		7.8±1.9

SD: standard deviation

The mean HbA1c in children (8.7±1.7%) was lower than in adolescents (9.5±2.2%) (p=0.05).

When evaluating the groups with HbA1c <7.5 and ≥7.5%, the one with better metabolic control had a lower score of daytime sleepiness compared to the group with altered HbA1c (11.6±3.2 versus 14.3±4.8; p=0.01) and longer sleep duration on weekdays (8.42±1.35 h versus 7.73±1.92 h; p=0.05). Despite this, there was no difference between groups in terms of sleep duration on weekend days, chronotype and social jet lag (Table 2).

**Table 2:** Sleep characteristics according to the serum concentration of glycated hemoglobin in children and adolescents. São Paulo, SP, Brazil, 2020.

	Glycated hemoglobin		p-value
	<7.5% (n=12)	≥7.5% (n=74)	
Pediatric Daytime Sleepiness Scale	11.6 (3.2)	14.3(4.8)	0.01*
Sleep duration on weekdays (h)	8.4 (1.4)	7.7 (1.9)	0.05*
Sleep duration on weekend days (h)	9.3 (1.5)	9.2 (1.5)	0.84
Social jet lag (min)	0.4 (0.7)	0.7 (0.9)	0.19
Chronotype	4.3 (0.6)	4.1 (0.8)	0.28

* p≤0.05 was considered statistically significant.

There was a negative correlation between age and sleep duration on weekdays (r=-0.39; p<0.01) and between age and chronotype (r=-0.38; p<0.01). In addition, there was a positive correlation between age and social jet lag (r=0.28; p<0.01).

Associated with glycemic control, the number of capillary blood glucose measurements had a positive correlation with sleep duration during the week (r=0.23; p=0.03) and with the chronotype (r=0.25; p=0.01); however, no correlations were found between the number of capillary blood glucose measurements and sleep duration on weekend days, between social jet lag and the PDSS score.

There was a negative correlation between HbA1c and sleep duration on weekdays (r=-0.23; p=0.03), but this correlation was not found on weekend days. Also, there was a negative correlation between HbA1c and chronotype (r=-0.21; p=0.05), and a positive correlation between HbA1c and social jet lag (r=0.27; p=0.01). Despite this, there was no correlation between the PDSS scores and HbA1c (Table 3).

**Table 3:** Correlations between glycated hemoglobin and sleep characteristics in children and adolescents. São Paulo, SP, Brazil, 2020.

Variables of sleep characteristics	r	p-value
Pediatric Daytime Sleepiness Scale	0.16	0.15
Sleep duration on weekdays	-0.23	0.03*
Sleep duration on weekend days	0.07	0.54
Social jet lag	0.27	0.01*
Chronotype	-0.21	0.05*

* p≤0.05 was considered statistically significant.

Prevalence of daytime sleepiness among patients with less than three years of T1DM diagnosis was higher when compared to those with three years or more of T1DM (57.1 versus 36.2%, respectively; p=0.03). In our sample, the type of insulin treatment, MDI or CSII, did not influence on daytime sleepiness (p=0.80).

The multiple logistic regression model was adopted to check the association between sleepiness and study variables such as: time since diagnosis of T1DM, HbA1c, sex, and age group. Patients with a diagnosis time less than three years were found to have 3.31 times more daytime sleepiness compared to patients with a longer time since diagnosis (95%CI [1.18–9.30]; p=0.02). In addition, patients with HbA1c≥7.5% were 6.43 times more likely to feel sleepy during the day when compared to patients with HbA1c<7.5% (95%CI [1.14–32.07]; p=0.04). These associations were maintained even after being adjusted for sex and age group, which indicates that the shortest time of diagnosis and the highest HbA1c were predictive factors for daytime sleepiness, regardless of sex and age (Table 4).

**Table 4:** Association between daytime sleepiness and study variables, according to adjustment variables: sex and age groups. São Paulo, SP, Brazil, 2020 *.

Study variabled		Daytime sleepiness	
		Raw	Adjusted
		OR (95%CI) p-value	OR (95%CI) p-value
Time of diagnosis			
	≥3 years	1	1
	<3 years	3.31 (1.18–9.30) 0.02	3.01 (1.04–8.66) 0.04
Glycated hemoglobin			
	<7.5%	1	1
	≥7.5%	6.04 (1.14–32.07) 0.04	6.43 (1.19–34.63) 0.03
Sex			
	Male	-	1
	Female		0.98 (0.38–2.47) 0.97
Age group			
	10–12 years	-	1
	13–18 years		0.58 (0.22–1.52) 0.28

* Multiple logistic regression model, with significance level of p<0.05%; OR: Odds Ratio; 95%CI: 95% confidence interval.

## DISCUSSION

Sleep characteristics are influenced by social, cultural and family models, as well as by psychological and biological factors related to the presence of T1DM.^14^ In this study, we identified important glycemic control factors with sleep characteristics, with associations between them. In T1DM patients, inadequate glycemic control is linked to poor-quality sleep, shorter duration sleep and obstructive sleep apnea.^5^ ^,^ ^15^ ^,^ ^16^

In our study, most patients had ended the “honeymoon” period, which is an early and transient phase of T1DM with less need for exogenous insulin (below 0.5 IU/kg/day), due to persistent insulin secretion by the remaining beta cells.^17^ They were also in puberty, with higher levels of growth hormones (GH), glycemia-counteracting hormone and, consequently, greater need for daily insulin for metabolic control.^18^ These factors may have influenced the mean HbA1c in the studied population. Although the HbA1c values are above the target currently recommended by the Brazilian Diabetes Society (<7%), this level corresponds to the average found in other reference services for T1DM.^19^

Most patients were being treated with T1DM, which requires several daily insulin applications, including nighttime, to correct glycemia and carbohydrates ingested at meals. In addition, a high frequency of capillary glycemic monitoring is required to control glycemia. Thus, treatment with Basal-Bolus insulins requires frequent adherence and monitoring by parents and the children/adolescents themselves.^20^ ^–^ ^22^

No difference was found in the prevalence of daytime sleepiness according to the type of insulin therapy (MDI or CSII). Both therapies are known to require strict glycemic controls and frequent self-care practices, which can impact patients’ sleep. Despite this, Sharifi et al.^23^ showed that adolescents using CSII reported less sleep disorders and longer sleep duration than those who were on MDI.

The mean sleep duration on weekend days was higher than on weekdays, as a way to compensate for the weekly sleep debt, giving rise to social jet lag. Comparing these data with those of Schnurbein et al.,^16^ the duration of sleep was longer on weekdays and shorter on weekends, probably because of social jet lag, cultural differences and different lifestyle habits between populations. As for the mean value of the chronotype, they were similar between both samples.

Previous studies conducted with young people showed results similar to ours when it comes to sleep duration. Frye et al.^22^ observed a sleep duration of 7.45±0.74 h in their population, with suboptimal glycemic control (mean HbA1c of 9.11±1.95%), which highlights the importance of addressing sleep time in the management of children and adolescents with T1DM.

Reutrakul et al.^5^ reported that children with T1DM have 26 minutes less sleep than controls, due to nocturnal hypoglycemia, night care and the higher frequency of polyuria, polydipsia and nocturia, especially in patients not well controlled.

According to Monzon et al.,^24^ the shorter the duration of sleep—due to a higher frequency of nocturnal awakenings, parental stress due to night care, concern about the disease and continuous monitoring—, the higher the levels of cortisol and worsening of glycemic control.

Perfect et al.^3^ reported longer sleep duration in patients with lower HbA1c, which is in line with our findings.

The PDSS is a pediatric daytime sleepiness scale in which the cutoff used is 15 points,^11^ with the detection of a significant percentage of daytime sleepiness (42.7%), and mean of 17.9±3.1 in our sample. Excessive daytime sleepiness is characterized by unsatisfactory levels of sleep, difficulty in staying alert during the day and increased subjective feeling of need for sleep. In addition, it is associated with low academic performance in children and adolescents.^11^ ^,^ ^25^

In our sample, morning patients had higher values of social jet lag, which can contribute to greater daytime sleepiness. Social jet lag is an irregularity in sleep pattern. It is a discrepancy between biological and social time, characterized as a conflict between the preference oriented by chronotype for hours of sleep and the time needed to fulfill social obligations, such as school and work.^26^ Perfect et al.^27^ reported that sleep debt in adolescents stems from their preferences for later hours of sleep and awakening, with maintenance of environmental demands, such as early school start times. In addition, adolescents tend to have a physiological delay in sleep rhythm due to changes in the homeostatic sleep process and sensitivity to light, which are stimulated by hormonal variations.^28^ Oliveira et al.^29^ determined that social jet lag is the variable with a protective potential for cardiometabolic risk, because of a possible compensatory mechanism, observed in the so-called recovery sleep present in the adolescent population.

We found a positive correlation between social jet lag and HbA1c in the present study, which was also detected by Perfect et al.^27^ in adults with T1DM and by Monzon et al.^24^ Thus, the greater the social jet lag, the greater the HbA1c.

Adolescents with T1DM tend to have social jet lag twice as high as adolescents without the disease and have worsening levels of HbA1c. In addition, changes in sleep patterns lead to lower glycemic control, as they infer a greater need for daily insulin in patients with increased social jet lag, due to increased insulin resistance and lower glycemic monitoring.^30^ Sleep variability in weekdays and weekends tends to increase insulin requirements, a fact confirmed by Schnurbein et al.^16^

In our study, higher levels of HbA1c were correlated with shorter sleep duration on weekdays, greater social jet lag and morning chronotype. In addition, high levels of HbA1c were associated with a greater chance of daytime sleepiness. An influencing factor in the correlation between glycemic control and sleep is the possibility of compensating for the negative effects of sleep restriction as insulin doses used increase.^16^

One of the most relevant findings in our study is the association between the shorter time since diagnosis of T1DM and higher levels of HbA1c and excessive daytime sleepiness, regardless of age and sex. Greater daytime sleepiness in patients with a shorter duration of T1DM and higher levels of HbA1c can be explained by the inexperience of caregivers with regard to blood glucose monitoring and insulin applications, the need for a greater number of capillary blood glucose measurements to adjust insulin therapy and the concern with the occurrence of nocturnal hypoglycemia, which leads to more frequent nocturnal awakenings.

A limitation of this study is the method of self-reported sleep characteristics. Although polysomnography is the gold standard for assessing sleep characteristics, the self-report questionnaire is the most economical and easiest method for population studies. Another limitation was the sample size, justified by its convenience. This study was partly carried out during the COVID-19 pandemic, a period in which some daily habits of children and adolescents were modified by the suspension of in-person classes. Despite this, the participants and guardians were instructed to answer the questionnaires according to habits prior to the pandemic period. Despite the limiting factors, this is one of the first studies conducted in a single Brazilian center with children and adolescents with T1DM that found an important association between PDSS, time of diagnosis and glycemic control, in addition to the evaluation of chronotype of that population.

We concluded that patients with higher HbA1c had greater daytime sleepiness, morning chronotype, shorter sleep duration on weekdays and greater social jet lag. We also observed that shorter diagnosis time for T1DM and HbA1c above 7.5% are associated with a greater chance of daytime sleepiness. To better understand the chronic effects of suboptimal glycemic control and its association with sleep characteristics and quality, further longitudinal studies in the pediatric population with this disease are needed.
